# Low Temperature Nitriding of Metal Alloys for Surface Mechanical Performance

**DOI:** 10.3390/ma16134704

**Published:** 2023-06-29

**Authors:** Michel Drouet, Eric Le Bourhis

**Affiliations:** Institut P’, CNRS, Université de Poitiers, Bd M. & P. Curie-TSA 41123, F-86073 Poitiers, France

**Keywords:** metal alloy, nitriding, plasma, implantation, gas, structure change, surface mechanical performance

## Abstract

Metallic alloys are, by essence, ductile and stiff and can support loads without sudden rupture. This ductility becomes a disadvantage when applications require wear resistance. In this case, the hardening of the surface is required while retaining a core performance. Here, nitriding at low temperatures has proven to be beneficial and has potential. In fact, any phase transitions or unwanted compound precipitations that occur at higher temperatures have to be avoided as they would have a deleterious effect on the chemical homogeneity and mechanical properties. The present contribution summarizes the achievements made with such treatments on metallic alloys. We considered the most popular treatments, namely plasma, implantation, and gas nitridings.

## 1. Introduction

Metals deform first elastically and then plastically over a threshold known as their yield strength. Hence, a metal is resilient contrary to a ceramic, which is subject to sudden brittle fracture [1]. This constitutes a structural advantage for metals compared to ceramics like alumina and zirconia when used, for example, as prostheses. So far, metals are less resistant to wear than ceramics, and the treatment of their surface is required for zones submitted to friction. A broad range of treatments has been used to harden metal surfaces, changing their structure and microstructure (steel quench) and introducing sub-surface dislocations (work hardening), residual stresses, atomic solutes, and precipitates. Alternatively, hard coatings can also be deposited onto metal surfaces. Whatever the chosen treatment, the main goal is to improve the surface wear resistance, usually by increasing the hardness, without jeopardizing the core properties of the alloy or other surface properties like corrosion resistance. Hence, low-temperature nitriding or carburizing is of particular relevance. The term ‘low-temperature’, when used by the community, refers to a temperature well below any phase transitions (β-transus temperature of the alpha phase in TA6V, for instance) or unwanted compound precipitations (nitrides in stainless steel, for instance) [2]. In fact, these structural changes could have a deleterious effect on the chemical homogeneity (chromium precipitation induces loss of corrosion resistance in stainless steels, for instance) and mechanical properties (stress concentration at precipitates). A full book would not suffice to cover all these treatments [2,3], and here, we focus on nitriding treatments that have been used successfully to modify metal alloys’ subsurfaces with spectacular hardness enhancement.

Nitriding can be achieved with a variety of techniques [3], the most popular ones being namely plasma, implantation, and gas or a combination of them (PBII) [4]. Nitriding methods have been applied to a number of different metals and alloys [5,6,7,8,9,10]. Below, we illustrate the mechanical strengthening achieved with steel and titanium alloys that have been the most successful. The hardening profile and depth can be substantially tailored and are, in fact, dependent on the processing conditions. Nitriding is a thermochemical treatment involving surface enrichment in nitrogen and its diffusion to the inside of the bulk. Therefore, structural changes are vast, with a number of different interaction possibilities between the nitrogen-rich atmosphere and the metal surface, namely adsorption, sputtering, and implantation [4]. The induced phenomena reported comprise the nitrogen expansion of the unit cell, a new phase creation, like nitrides or alphagen phase transformation in titanium, for example, precipitation, grain rotations, and swelling. The benefits in terms of mechanical performance are discussed, as there is a close link between structural and surface mechanical modifications.

So far, such studies have been difficult since the generated subsurface was highly heterogeneous. Considering diffusion only, nitrogen atom mobility was thermally activated and, hence, limited as the used temperatures were to be chosen so that the initial core performance of the metal alloys could be retained. As a consequence, the structure and mechanical response changed very rapidly at the subsurface to reach the core ones. Hence, dedicated tools had to be considered to detect and measure the rapidly changing structure and properties.

The present article will start with an overview of the techniques employed for nitriding of metal alloys (Section 2), then discuss the subsurface structure and mechanical performance obtained (Section 3) before envisioning prospects in the field (Section 4).

## 2. Nitriding with Plasma, Implantation and Gas

### 2.1. Plasma Nitriding

Plasma nitriding is the most used technique compared to implantation and gas. It is carried out at a reduced pressure with plasma maintained using a voltage or an electromagnetic wave. Plasma nitriding is widely used for metal surface treatments on an industrial scale. It allows for the treatment of pieces that have a rather complex shape with a few limitations in shape ratio (high l/d ratio holes) or edge sharpness (Table 1). Although atmospheric pressure plasma techniques have been developed during the last decade [11], most of them use low-pressure plasmas [12]. The main technologies are the pulsed DC plasma [13], Radio Frequency driven plasmas (Inductively Coupled Plasma: ICP, Antenna generated or less common Capacitive or Surface Wave Coupling), usually working in a post-discharge mode, and microwave-based ECR (Electron Cyclotron Resonance) plasmas. Whatever the technology, the main advantage of the plasma atmosphere for surface treatments is the presence of highly reactive species: ions and radicals [14]. These species play a central role in the incorporation process of nitrogen into the material [15]. Very often (if not in most cases), a certain amount of hydrogen is also introduced into the plasma gas mixture. The hydrogen (H^+^ and H_2_^+^) and N_x_H_y_^+^ species contained in the plasma are very efficient for reducing oxide layers that are present on the surface of metallic pieces. In these processes, the plasma mainly acts as a surface concentration booster; the scale thickness and the diffusion length are still governed by the thermodynamic parameters, which is the treatment temperature in the first place.

### 2.2. Implantation Nitriding

The implantation process starts with the generation of ions in a chamber where the gas is excited by electrons emitted from a cathode. The generated ions are extracted by an electric field (voltage) and are selected with a magnetic field (magnetic filter) in terms of their nature and energy. The ion beam is later accelerated in the range of 10–500 keV to the metallic piece to be treated. As the ions penetrate the subsurface, they progressively lose their energy and generate an implanted sublayer. The N concentration distribution is Gaussian-like with a maximum located between a few tens of nanometers to a micrometer beneath the surface (at the so-called “stopping range”); depending on the ion’s energy, a great amount of implantation defects can also be created between the surface and the N distribution. Therefore, when the implantation is operated at an elevated temperature (500 °C and above for a titanium alloy, for instance), N diffusion can be considered both due to the surface and the inside of the material; these two directions may have different diffusion coefficients because of their implantation defects [9]. Of course, when diffusion allows for a deeper region to be treated, this implantation is diluted. It is important to note that high doses require long implantation times with the locally induced heating of the specimen, which can be deleterious because of the relaxation of residual stresses and grain growth. Surface sputtering also occurs at the material surface [16], lowering the nitrogen retained dose.

### 2.3. Gas Nitriding

Gas nitriding [17,18,19] (also known as conventional nitriding) requires a furnace with controlled pressure and an atmosphere containing N_2_ or NH_3_ molecules. The piece to be treated is introduced into the furnace. As expected, temperature plays an important, not to say a central, role in both surface reactions and diffusion. An activation surface treatment may be needed at a low temperature to allow for an efficient enough reaction [18,20]. Concurrently, activating diffusion may again affect the original core metal, and this has pushed toward other techniques like plasma and implantation (above sections) or a combination of both (next section). It is also important to note that the presence of hydrogen in the atmosphere has been shown to be important for the performance of this process, and this has become a common practice when using the different nitriding techniques described here.

### 2.4. Plasma Based Ion Implantation (PBII) Nitriding

Plasma-based ion implantation (PBII) combines the plasma (Section 2.1) and implantation (Section 2.2) and hence their respective advantages and drawbacks (Table 1). When it was developed in the 1980th [21], the main goal of PBII (often referred to as PIII, Plasma Immersion Ion Implantation) was to combine the benefits of both ion implantation (deep insertion of species) and the versatility of plasma treatments, e.g., by overcoming the line-of-sight restriction, for metallurgical applications [22,23,24]. Although interesting laboratory results have been obtained in different domains [25,26,27,28], the technique remains quite confidential as the inherent difficulties to upscale are not compensated by the benefits obtained compared to standard plasma treatments.

## 3. Surface Mechanical Benefits

### 3.1. General Features and Scales

**Figure 1:**
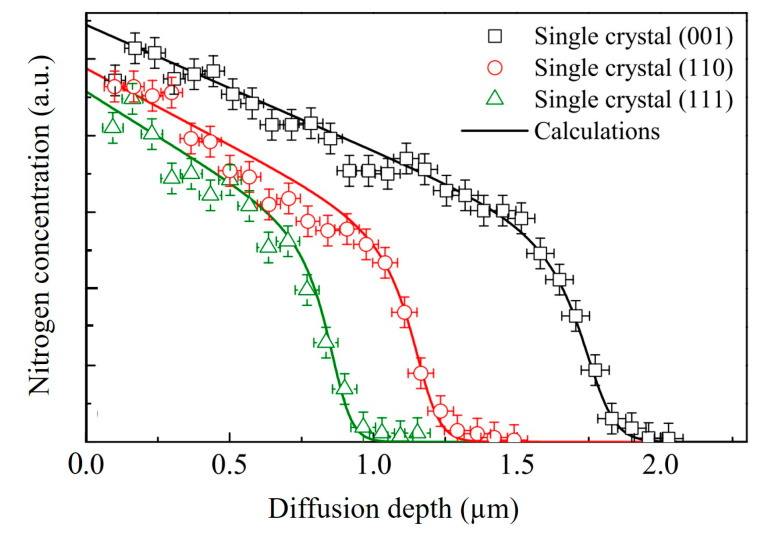
Experimental nitrogen distribution profiles of nitrided single crystals with different orientations (symbols) and corresponding calculated profiles (lines) according to the trapping-detrapping model (adapted from [34]).

**Figure 2:**
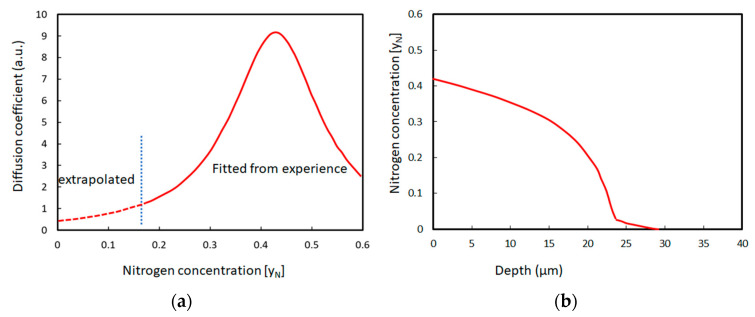
Concentration dependent diffusion coefficient (**a**) and typically calculated nitrogen depth profile (**b**) (adapted from [38]).

Although the exact nitrogen concentration, in this case, could, to some extent, depend on the nitriding technique, the general profile characteristics are mainly determined by the treated material type. For most low or medium-alloy steels, nitriding results in the formation of an iron nitride layer compound followed by a nitrogen diffusion profile. Two types of nitrides could be present: the γ′-Fe_4_N or/and the ε-Fe_2-3_N [7]. Marot et al. [29] pointed out the parabolic diffusion behavior and the absence of a nitride surface layer (white layer) in the case of low-temperature plasma nitriding at the floating potential. The nitrogen in excess of the solubility limit forms “sub micron” nitride precipitates. On the contrary, Zagonel et al. mentioned the existence of an ε-Fe_2-3_N layer, even at a low temperature, for pulsed plasma nitriding [30]. Nitride precipitates are, however, present underneath this layer and can account for most of the hardness improvement. The discrepancy between these two experiments could be explained by the influence of the involved sputtering rate (energy of the ions) [31], as pointed out on an M2 tool steel by Mohammadzadeh et al. [7]. Stainless steels, and more generally, austenitic alloys, present a very specific and uncommon nitrogen profile (Figure 1) [32,33,34,35,36]. This characteristic profile results from a non-conventional diffusion process that is governed by a strong affinity between chromium and nitrogen. A model where part of the nitrogen atoms are “trapped” in the vicinity of Cr atoms until the saturation of the traps was proposed by Möller [37]. The dependence of the flux upon the local concentration is responsible for the obtained profile. This was formalized by Christiansen et al. [38] using a concentration (octahedral sites occupancy y_N_) dependent diffusion coefficient (Figure 2) together with the solubility product of Cr and N in the γ phase. In these alloys, the γ phase could accommodate up to 25 at% of nitrogen and form the so-called expanded austenite, usually referred to as γ_N_ or the S phase.

**Figure 3:**
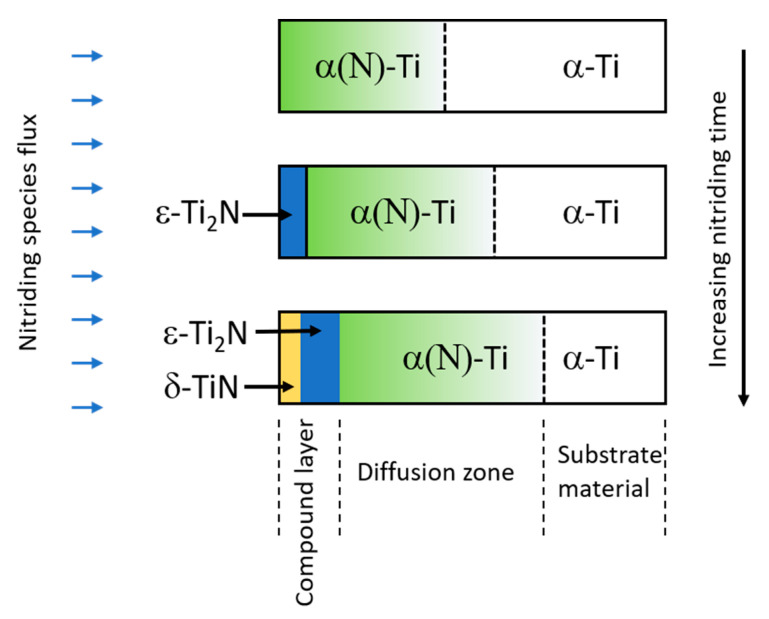
Schematic model for titanium nitriding process (adapted from [39]).

As regards titanium and titanium alloys, the nitriding process has been well modeled by Zhecheva (Figure 3) [39]. According to this model, nitrogen was first incorporated into the material until it reached a surface concentration of 22 at% (maximum solubility in αTi), followed by a diffusion profile. An additional nitrogen incorporation induced a phase transformation to ε-Ti_2_N followed by a second phase transformation, leading to δ-TiN. The resulting structure is a dual, very hard compound layer supported by a diffusion zone. It has to be noticed that, as nitrogen is an alphagen element, in the case of mixed α-β or β-alloys, this process is accompanied by the drastic phase transformation of the titanium itself.

Aluminum and its alloys are very difficult to nitride. Only high-power plasma combined with the polarization of the sample and conducted at a high temperature (>500 °C) leads to the formation of a significant ratio of aluminum nitride AlN in the mixed AlN/Al_2_O_3_ surface layer with very little diffusion [23,40].

As discussed above, the conditions used for nitriding rule the depth and the extent of the modification of the sub-surface. Accordingly, different scales have been considered to assess the gain in mechanical performance. Authors have been used extensively in both conventional micro-indentation [41,42,43] as well as instrumented nanoindentation [44]. It is important to take caution when comparing micro- and nano-indentation values as different tested volumes are considered. So far, a key point to be kept in mind is that roughness can be generated during the treatment of metallic polycrystals because of the orientation-dependent diffusion rate and swelling, as well as grain rotation [45,46]. The advantage of conventional micro indentation is that it is less affected by generated roughness and can be used on the treated surface as well as on prepared cross-sections. Instead, nanoindentation requires cross-sections to be prepared or the polishing of the treated surface with the risk of losing the most superficial material [47]. So far, the strong mechanical gradient at the subsurface makes such preparations difficult [25]. Nonetheless, both cross-sectional and surface nanoindentation testing have been realized on nitrided specimens. In fact, surface preparation could also be used to etch the treated material progressively and realize nano-mechanical tomography, for example, on 316L stainless steel in [47].

### 3.2. Elastic-Plastic Response and Wear

**Figure 4:**
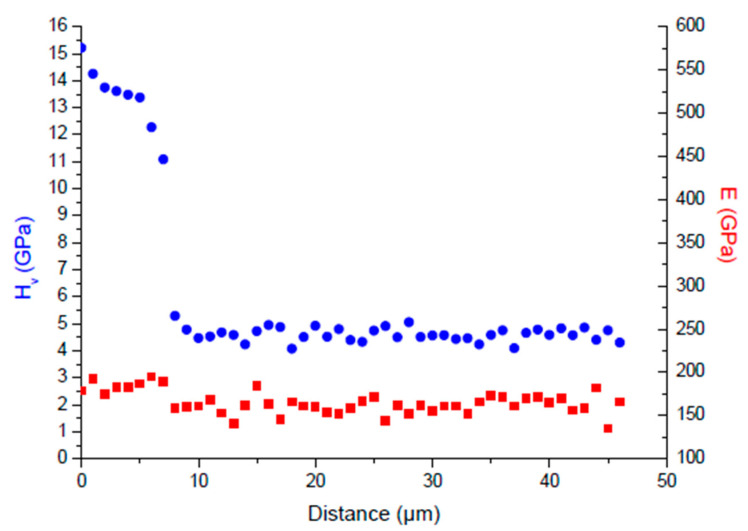
Hardness (circles) and indentation modulus E (squares) obtained on nitrided stainless steel (nanoindentation measurement on a cross-section, the treated surface is on the left-hand side of the graph, adapted from [48]).

Instrumented nanoindentation offers the possibility to assess both elastic and plastic changes in the subsurface by analyzing loading–unloading curves. Instead, a conventional micro indentation can be used only to assess the plastic response [42,43] unless the geometrical consideration of the Knoop mark can be considered. Most commonly, a sharp diamond indenter is used with a Vickers or Berkovich geometry (four-sided pyramid and three-sided, respectively). Importantly, it has been observed in several alloys that plastic and elastic responses are not affected similarly by nitriding treatment, and this emphasizes the importance of using instrumented nanoindentation to assess both responses. For instance, a PBII nitrided 304 L stainless steel showed drastic changes in terms of Hardness (with an almost three-fold increase at the surface, Figure 4), while the indentation modulus was almost retained [48]. This phenomenon is fortunate as Archard’s law reveals that wear decreases as hardness increases for a given family of materials. In fact, an increase in both hardness and wear performance has been reported in nitrided titanium alloy [49]. More recently, the elastic-plastic ratio was considered more appropriate as an indicator of wear performance. It was also observed to increase accordingly. It can be noted that, depending on the authors, the elastic-plastic ratio can take different forms such as H/E, H^3^/E^2^ [1]; in any case, wear performance can be improved with an increase in the hardness and a moderate change in the elastic modulus.

### 3.3. Surface Mechanical Performance

**Figure 5:**
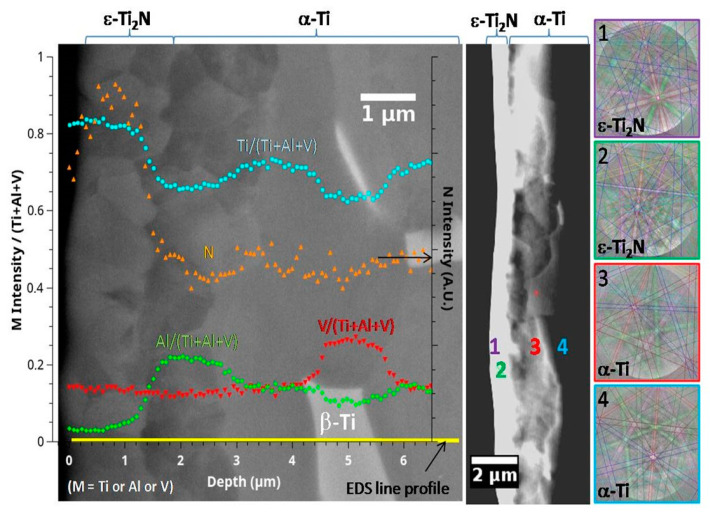
SEM, EDS and EBSD results obtained on a cross section of the Ti-6Al-4V sample treated by PBII (25 kV, 200 Hz, 10 μs) for 240 min at 800 °C in a mixture of 90% N_2_—10% H_2_ at 1 Pa [26].

Surface mechanical performance can be closely related to the structural changes induced by the nitriding process as well as to the parameters used. This offers a large range of performance. For instance, for the gas nitriding of Ti in a relatively broad range of temperatures and durations (700–950 °C and up to 16 h), surface hardness was reported with a two-to-three-fold increase [50]. The 3D plots allowed us to observe the interplay between temperature and time [50]. So far, many different phenomena comprising the nitrogen expansion of the unit cell, precipitation, grain rotations, in-depth diffusion, and alphagen element-induced phase transformation (in Titanium alloys) have to be considered. Of course, the parameter of first importance was nitrogen concentration which can be estimated from the glow discharge optical emission spectroscopy (GDOES) and Energy Dispersive Spectroscopy (EDS) analyses in the scanning electron microscope (SEM) (Figure 5). Transmission electron microscopy (TEM) offers further insights into the structural changes that happen during the treatment [27] and an outlook for the future (Section 4).

The research has been market-driven, with demands for nuclear plants, aeronautic components as well as medical prostheses. Nitriding treatments have been applied mostly to steels and titanium alloys (Table 2). It can be noticed that, although efficient, the nitriding treatment becomes ineffective for superalloys since the pieces are set at a temperature above their process temperature.

The nitriding treatment of Ferritic steels yields surface nitrides, γ′-Fe_4_N, with a small volume fraction of ε-Fe_3_N/Fe_2_N, which is beneficial for hardening and wear performance [7,51]. On the other hand, stainless steel surfaces show expanded austenite after nitriding treatment [2], with a strong enhancement of hardness, while the elastic response is almost retained [47]. Wear performance can also be improved as well as expected from Section 3.1 [2]. Nitrides δ-TiN/ε-Ti_2_N are also formed in Titanium alloys and in the depth of an α-Ti(N) solid solution (Figure 2) [26]. These latter structural changes are accompanied by a two-fold increase in the hardness while, as for steel, the elastic response remains almost unaffected.

**Figure 6:**
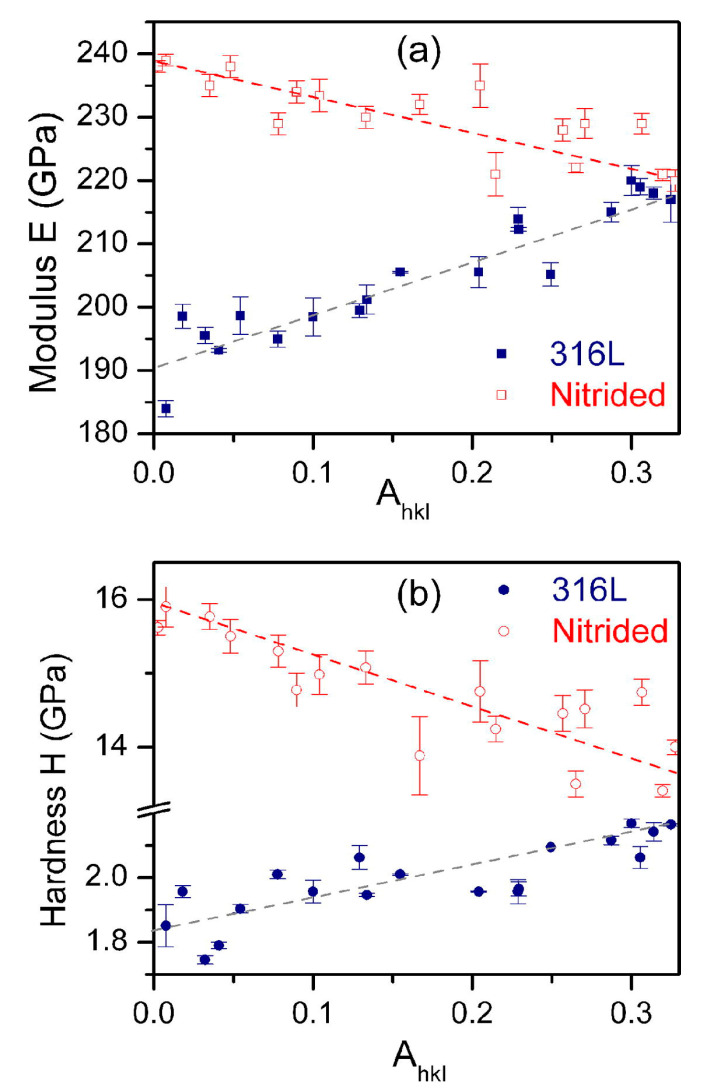
Indentation modulus (**a**) and hardness (**b**) as a function of the anisotropy factor A_hkl_ for untreated and nitrided 316L. Dotted lines are guides for the eyes and the bars correspond to the standard deviation. Reprinted from [52] with permission from Elsevier.

Nanoindentation offers the possibility to assess the direction-dependent mechanical changes of polycrystalline materials once the testing conditions are adjusted to confine the probed volume to a single grain. It has been reported that the nitriding treatment affects this dependence in stainless steel (316L) dramatically, either in terms of the elastic or plastic response [52,54]. Indeed, Figure 6 plots these dependencies as a function of the anisotropy factor defined as A_hkl_ = (h^2^k^2^ + k^2^l^2^ + l^2^h^2^)^2^/(h^2^ + k^2^ + l^2^)^2^, h, k and l which are the Miller indices of the orientation. It is important to note an inversion to both hardness and modulus dependence after the nitriding treatment. Kücükyildiz and co-workers [54] attributed this behavior to the presence of a certain amount of oversaturated γ’_𝑁_ (M_4_N_1+x_) nitride in the uppermost nitrided layer. In the latter case, gas nitriding was used in single crystals. It revealed that nanoindentation could be used at the scale of a grain in such polycrystals. Moreover, mechanical tomography (see also Section 3.1) could be carried out with a higher depth resolution [54]. Again, these induced changes were more important in terms of hardness compared to the modulus.

## 4. Conclusions and Prospects

The nitriding treatments reviewed here have proved very successful in hardening metal surfaces while retaining their core structure. This strategy allows for improving surface resistance and structural integrity as long as the pieces are not set at a temperature above the treatment temperature [19,53,55,56,57,58,59,60,61]. Hence, while nitriding has been extensively operated on steels and titanium alloys, as illustrated here, superalloy treatments, although efficient, are less interesting as most aeronautic applications use high temperatures [35,36,59]. Since the early work of Bell and co-workers, the low-temperature nitriding of different metallic alloys has been continuously improved, both from an experimental point of view and theoretical aspects. Among these, stainless steel nitriding has been extensively studied during the last three or four decades and can be considered one of the most successful applications of low-temperature conditions.

The nitriding approach has also proved a great advantage over ceramics when prostheses are considered since structural integrity is of the utmost importance. In fact, metal pieces with complex shapes can be treated. Moreover, complex-shaped metal components are much cheaper than their equivalent ceramic ones. In this applicative domain, titanium alloys are by far the most interesting choice and can give rise to a tremendous amount of research (see, for example, [3,8,25,26,39,42,43,50,62]).

Still, improvements and prospects are expected in the near future, with an observed large activity in the field [63,64,65,66,67,68,69,70]. Overall, a more detailed picture of the complex nitriding process could be obtained. It has been revealed that the low-temperature nitrided layer characteristics of ferritic stainless steel are highly initial-microstructure dependent, and this is to be considered carefully when targeting a particular performance [63]. Attention can also be paid to the absorbed H_2_ in the nitrided specimen when treated under a different H_2_ proportion because of the consequent deleterious influence [65], while depth and time-resolved data using in situ XRD can allow the initial expanded austenite formation to be captured. Different innovations can allow further incremental improvements. For instance, previously to nitriding, some authors have deposited coatings that form harder and corrosion-resistant phases upon the treatment [71]. Hard coatings can also be added to the nitrided pieces [72,73], although care has to be taken with the roughness induced by the nitriding process. Plasma nitriding with the titanium addition could allow a thinner brittle compound layer and a thicker ductile effective hardening layer to be produced on the surface of steel [74].

Moreover, it is of interest to deepen and smooth the mechanical gradient so as to avoid the ‘egg effect’ or a too-large mechanical contrast between the surface and the core. Combined treatments comprising plasma nitriding plus oxidation, for instance, are being proposed and are effective in smoothing the mechanical gradient while also deepening the influence of the treatment [42,43,75,76].

A further understanding of induced structural-mechanical changes at the scale of the grains is also required, and in situ, local techniques could be of the utmost importance since the mechanical response of individual grains can be assessed [77,78,79,80].

## Figures and Tables

**Table 1:** Overview of nitriding treatments.

Technique	Advantage	Drawback	Notes
Plasma	Moderate temperature, some complexity of the shape acceptable: lower gas consumption and less waste	High l/d ratio holes not well treated, sputtering, possible edge effects	
Implantation	Low temperature, overcomes surface barriers: extremely low gas consumption and waste	Mainly Planar treatment	Convex shapes possible but with complex experimental sets
Gas	Easy complex shape treatments	High temperature	High l/d ratio holes easy

**Table 2:** Structural induced changes in surface mechanical performance.

Metallic System	Nitriding Treatment	Structural Change	Surface Mechanical Performance
Ferritic steel	Gas (550–650 °C)	Compound layer with cracks γ′-Fe_4_N ε-Fe_3_N/Fe_2_N nitrides and diffusion layer	2–4 fold increase in surface hardness [17]
Ferritic steel	Plasma (450–560 °C)	γ′-Fe_4_N and ε-Fe_3_N/Fe_2_N nitrides and diffusion layer γ′-Fe_4_N micro precipitates	2–3 fold increase in surface hardness [51] and refs therein [29]
Austenite stainless steel	Gas (430–450 °C)	Expanded austenite or γ_𝑁_ phase	3 fold increase in surface hardness, limited elastic change [18]
Austenite stainless steel	Plasma (<430 °C)	Expanded austenite or or γ_𝑁_ phase	3 fold increase in surface hardness, limited elastic change [48,52]
Austenite stainless steel	PBII (430 °C)	Expanded austenite or or γ_𝑁_ phase	3 fold increase in surface hardness, limited elastic change [23]
Titanium alloys	Gas (700–950 °C)	Formation of δ-TiN and ε-Ti_2_N High temperature enhanced in-depth diffusion	2–3 fold increase in surface hardness up to 17 GPa [50] In-depth hardness gradient [53]
Titanium alloys	Plasma (650–850 °C)	Formation of δ-TiN/ε-Ti_2_N, in the depth α-Ti(N) solid solution	2 fold increase in surface hardness, limited elastic change [39]
Titanium alloys	PBII (500–800 °C)	Formation of δ-TiN/ε-Ti_2_N, in the depth α-Ti(N) solid solution nitrides formed at low temperature (500 °C)	2 fold increase in surface hardness, limited elastic change [9,26]

## Data Availability

The data presented in this study are available on request from the corresponding author.
